# Phosphatases in the cellular response to DNA damage

**DOI:** 10.1186/1478-811X-8-27

**Published:** 2010-09-22

**Authors:** Alyson K Freeman, Alvaro NA Monteiro

**Affiliations:** 1 Risk Assessment, Detection, and Intervention Program, H. Lee Moffitt Cancer Center and Research Institute, Tampa, Florida, 33612, USA; 2 University of South Florida Cancer Biology PhD Program, Tampa, Florida 33612, USA; 3 Present address: NCI-Frederick, P.O.Box B, Building 560, Mailstop 17, Frederick, MD 21702 USA

## Abstract

In the last fifteen years, rapid progress has been made in delineating the cellular response to DNA damage. The DNA damage response network is composed of a large number of proteins with different functions that detect and signal the presence of DNA damage in order to coordinate DNA repair with a variety of cellular processes, notably cell cycle progression. This signal, which radiates from the chromatin template, is driven primarily by phosphorylation events, mainly on serine and threonine residues. While we have accumulated detailed information about kinases and their substrates our understanding of the role of phosphatases in the DNA damage response is still preliminary. Identifying the phosphatases and their regulation will be instrumental to obtain a complete picture of the dynamics of the DNA damage response. Here we give an overview of the DNA damage response in mammalian cells and then review the data on the role of different phosphatases and discuss their biological relevance.

## Introduction

Maintenance of genomic integrity is an essential part of cellular physiology. Genotoxic insults that induce DNA breaks must be repaired in order to prevent the propagation of mutations that can contribute to malignant transformation. DNA damage occurs following a variety of stimuli including ionizing radiation (IR), ultraviolet radiation (UV), replication stress, chemicals from the environment, and reactive oxygen species that are produced as a byproduct of cellular metabolism.

**Figure 1:**
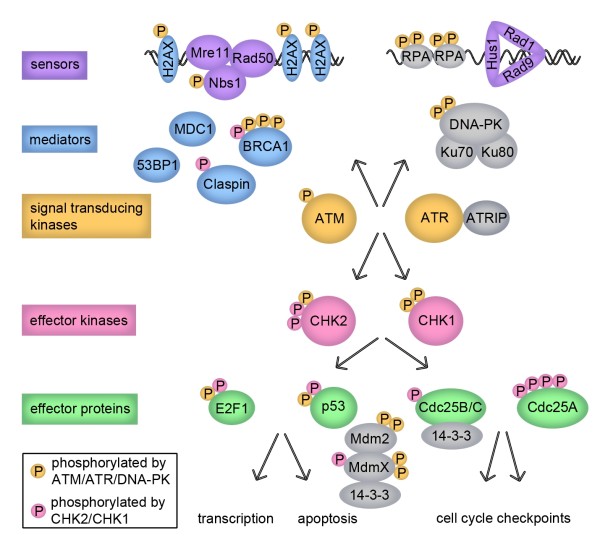
**A simplified view of the cellular response to DNA damage**. Single and double stranded DNA breaks signal through the sensors (MRN and 9-1-1) shown in purple, mediators (H2AX, BRCA1, MDC1, 53BP1) shown in blue, signal transducing kinases (ATM, ATR) shown in yellow, effector kinases (CHK2, CHK1) shown in pink, and effector proteins (E2F1, p53, Cdc25) shown in green, leading to gene transcription, apoptosis, and cell cycle arrest. Proteins that are phosphorylated by ATM, ATR, and/or DNA-PK are marked by a yellow phosphate group and proteins that are phosphorylated by CHK2 and/or CHK1 are marked by a pink phosphate group.

The processes by which cells repair damage to DNA and coordinate repair with cell cycle progression are collectively known as the DNA damage response (DDR). In cases in which the damage cannot be repaired, prolonged cell cycle arrest can lead to senescence or the induction of apoptotic signals [1-3]. Signaling through the DDR occurs through a series of distinct but interconnected pathways that are better visualized as a network [4]. This network includes proteins that have been classified as sensors, signal transducing proteins, effector kinases, mediators, and effector proteins (Fig. 1)[1]. Although this classification is arbitrary and the distinction is sometimes blurred, it facilitates our global understanding of the information flow in the network.

Analogous to growth factor receptor signaling, DNA damage signaling is also driven primarily by changes in protein localization and post-translational modifications. Among post-translational modifications, serine and threonine phosphorylations occupy central stage (Additional file 1, Table 1). Although there has been significant progress in our understanding of the role of these phosphorylations and the regulation of DDR kinases [5], our knowledge of the role of dephosphorylations and phosphatase regulation in this context is still rudimentary. Here we provide an overview of DDR signaling and then discuss recent work that sheds light on how phosphatases are critical to the fine regulation of the DDR.

## Sensing the damage and activating the initial response

DNA damage is recognized by sensor proteins that initiate the activation of the DDR on chromatin. These sensors include the Mre11-Rad50-Nbs1 (MRN) and the Rad9-Rad1-Hus1 (9-1-1) complexes that localize to double stranded breaks (DSBs) or regions of replication stress and single stranded breaks, respectively [6,7](Fig. 1). Mre11 binds to Nbs1, DNA, and Rad50 and possesses DNA exonuclease, endonuclease, and unwinding activities [8]. While Rad50 may function to keep the broken ends of the DNA together, Nbs1 functions to recruit signal transducing kinases to the break site and mediates the DDR signal [9]. The structure of the 9-1-1 complex resembles the proliferating cell nuclear antigen (PCNA) sliding clamp that is loaded onto DNA at points of replication [6]. The Rad17-replication factor C (RFC) complex acts as the 9-1-1 clamp loader in a process analogous to RFC acting as the clamp loader for PCNA [10]. In a process that is not fully understood, localization of the MRN and 9-1-1 complexes to the sites of DNA damage in chromatin signals to activate the signal transducing kinases Ataxia-telangiectasia mutated (ATM), the ATM and Rad3-related (ATR) kinase, and the DNA-dependent protein kinase (DNA-PK), which are members of the phosphoinositide 3-kinase related kinase family.

Primarily in response to DSB, ATM dissociates from inactive dimers into active monomers [11]. Elegant biochemical and cell biological experiments led to the idea that ATM autophosphorylation at S1981 caused dissociation and was intimately linked to initiation of kinase activity [11]. However, recent mouse studies have shown that phosphorylation at S1981 is not required for ATM activation in vivo [12,13]. Rather, it seems to be required for the retention of ATM at the DSB sites through association with the mediator MDC1 [14]. Nbs1 seemingly acts both upstream and downstream of ATM, reinforcing the notion of a network rather than a linear pathway. ATM is known to phosphorylate Nbs1 on S343 and at the same time, Nbs1 and the MRN complex are required for full activation of ATM [9,15,16]. Along similar lines, the localization of ATR to the break site and its subsequent activation is dependent upon the 9-1-1 complex, binding between ATR and ATR-interacting protein (ATRIP), and replication protein A (RPA). RPA coats single-strand DNA and consists of 3 subunits: RPA70, RPA32, and RPA14 [17]. RPA32 is phosphorylated at T21 and S33 by ATM, ATR, and DNA-PK [18].

DNA-PK is part of the phosphoinositide 3-kinase related kinase family with ATM and ATR and is activated upon association with DNA [19]. DNA-PK is comprised of a catalytic subunit, DNA-PKcs, and the targeting subunit, the Ku70-Ku80 heterodimer [19]. The primary role of DNA-PK is to initiate non-homologous end joining to repair DNA DSB [20]. Two major autophosphorylation clusters in DNA-PKcs (referred to as ABCDE and PQR) regulate the ability of DNA-PK to function in DNA end processing during repair although the exact role of these phosphorylation events is not yet known [20]. Finally, recent proteomics approaches in yeast and mammalian cells have greatly expanded the list of potential substrates of kinases involved in the DNA damage response [21,22].

## Effector kinases and mediators: transducing the signal

The signal transducing kinases ATM and ATR signal through the effector kinases CHK1 and CHK2 (checkpoint kinase 1 and checkpoint kinase 2), which sustain and amplify the DDR signal [23]. Importantly, CHK1 and CHK2 are highly mobile messengers that are not restricted to chromatin compartments and are thus able to relay the message from complexes formed at or near breaks to other cellular substrates [24]. CHK2 is activated primarily in response to DSB through the phosphorylation of T68 by ATM [25-27] and subsequent oligomerization and autophosphorylation at T383 and T387 [28,29]. CHK1 is active even in unperturbed cells, but is further activated through the phosphorylation of S317 and S345 by ATR, primarily in response to single stranded breaks and replication stress [23].

The relative contributions of the effector kinases in development have been highlighted using mouse models. While *Chk2*-deficient mice are viable and do not have an increased risk for cancer, *Chk1*-deficient mice are embryonic lethal [30-33]. Although it is difficult to pinpoint which activities of CHK1 might be essential it is conceivable that CHK1 acts as the "workhorse" in responding to replication stress, which likely occurs in every cell cycle, while CHK2 functions in an inducible fashion after DNA damage caused by other stimuli. CHK1 also has important functions during normal cell division, as it was found to associate with centrosomes in interphase and regulate their separation [34].

Several mediator proteins such as H2AX, BRCA1, MDC1, Claspin, and 53BP1 work to coordinate the localization of various factors in the DDR, promote their activation, and regulate substrate accessibility [35]. The histone variant H2AX is phosphorylated by ATM, ATR, and DNA-PK on S139 upon DNA damage and this phosphorylated form is also known as γ-H2AX [36]. γ-H2AX forms nuclear foci visible through immunofluorescence that are both proximal and distal to the DSB site and is considered a marker for the presence of DSB [37]. γ-H2AX is required for the efficient retention of Nbs1 and the other mediator proteins BRCA1, MDC1, and 53BP1 at damage-induced foci [38-44].

BRCA1 is a target of multiple phosphorylations although for many of them the biological significance is largely unclear (reviewed in [45])(Table 1). BRCA1 S1387 and S1423 are targets of phosphorylation by ATM and these phosphorylations are required for the intra-S and G2/M checkpoints, respectively [46-48](Table 1). The S1387 site can also be phosphorylated by ATR and DNA-PK [49] perhaps ensuring a tight control of the intra-S checkpoint. The effector kinase CHK2 can phosphorylate BRCA1 on S988 in response to IR [50-52] but the functional consequences are still not clear. In response to UV, BRCA1 is also phosphorylated by ATR on S1457 [53]. Phosphorylated BRCA1 forms distinct nuclear foci that co-localize with γ-H2AX, MDC1, and the MRN complex. The role of BRCA1 in the complex seems to be to coordinate repair through both non-homologous end joining and homology-directed recombination [54,55].

MDC1 is phosphorylated in an ATM- and CHK2-dependent manner, and its BRCT domain directly recognizes the phosphoserine 139 in the carboxy end of γ-H2AX (Table 1) [42,56]. Recruitment of MDC1 to γ-H2AX foci is required for the formation of MRN, BRCA1, and 53BP1 foci [39,42,57]. Thus, MDC1 functions as a molecular scaffold to mediate parts of the DDR downstream of foci formation [58]. Claspin is a major regulator of the activity of CHK1 and binds DNA with high affinity. After DNA damage or replication stress Claspin is phosphorylated by CHK1, an event that is required for a Claspin-CHK1 interaction and subsequent full activation of CHK1 by ATR, in another example of the network nature of the DDR [59-63].

## Effector proteins: control of cell cycle progression

The effector proteins Cdc25, p53, and E2F1 function to activate cell cycle checkpoints and regulate the transcription of genes whose products are important in the end result of the DDR, whether it be DNA repair, apoptosis, or senescence.

The Cdc25 phosphatases regulate cell cycle progression by removing inhibitory Y15 phosphorylations from the cyclin-dependent kinases Cdk1 and Cdk2 [64]. The negative regulation of Cdc25 leads to the activation of G1/S, intra-S, and G2/M checkpoints. CHK1 phosphorylates Cdc25A on S76, leading to Cdc25A ubiquitination and degradation [65]. CHK1 and CHK2 also phosphorylate Cdc25A on S123, S178, S278, and S292 which leads to its IR-induced degradation [66]. CHK1 and CHK2 phosphorylate Cdc25C on S216 which allows 14-3-3 binding and subsequent sequestration of Cdc25C in the cytoplasm, away from substrates. Cdc25B is phosphorylated on S309, leading to inactivation through the same 14-3-3-mediated mechanism [64,67,68].

P53 is stabilized after DNA damage which allows it to activate the transcription of genes whose products participate in cell cycle arrest, DNA repair, senescence, or apoptosis, depending upon the stimulus [69]. Mdm2 negatively regulates p53 through ubiquitin-mediated proteosomal degradation [70-72]. MdmX also negatively regulates p53 by binding to Mdm2 and enhancing Mdm2 binding to and ubiquitination of p53 [73-75]. ATM, ATR, and DNA-PK phosphorylate p53 on S15, while CHK1 and CHK2 phosphorylate p53 on S20 which stabilize p53 by preventing binding to Mdm2 [76-78]. ATM and ATR also directly inhibit Mdm2 by phosphorylating it on S395 and S407, respectively [79,80]. ATM phosphorylates MdmX on S403 which leads to its ubiquitination and degradation [81] whereas CHK2 phosphorylates MdmX on S367 and S342 which promotes 14-3-3 binding and degradation of MdmX [82,83]. Therefore, multiple DDR proteins coordinate to stabilize and activate p53 through phosphorylation of p53 and its negative regulators Mdm2 and MdmX.

The transcription factor E2F1 is also activated by phosphorylation in response to DNA damage. ATM and CHK2 phosphorylate E2F1 on S31 and S364, respectively, which leads to E2F stabilization [84,85]. Accumulation of E2F1 leads to increased transcription activation and apoptosis, although the specific genes that are responsible for these cellular effects have not yet been determined [84,85].

## Fine-tuning the DDR: role of serine/threonine phosphatases

A simplistic reading of the scenarios described above suggests a unidirectional wave of phosphorylation events radiating from the site of damage that is progressively amplified to relay the signal to a large number of substrates in the cell (Fig. 1). Unfortunately, the biological relevance of many of these phosphorylated events is still unknown. These phosphorylated linear motifs are mainly recognized by 14-3-3 proteins, and by FHA and BRCT modular domains (Additional file 1, Table 1)[58]. We have little information about the dynamics of this system and although at first approximation it might seem a bistable system (stable "on" or "off" states), recent data have suggested the upstream kinases such as ATM and CHK2 signal in pulses [86,87]. Batchelor et al. experiments suggest that these pulses arise from periodic examinations of the status of the damage by ATM [86]. In any event, it is clear that fine-tuning of the response depends on the activity of phosphatases in order to prevent illegitimate activation of the DDR in the absence of damage as well as to allow rapid cessation of the signal once DNA is repaired.

**Figure 2:**
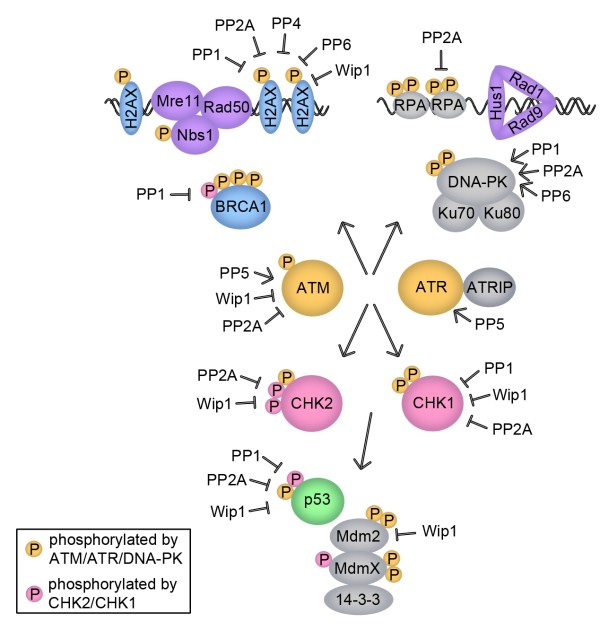
**Protein serine/threonine phosphatases in the DNA damage response pathway**. The positive (arrows) and negative regulation of DDR proteins via protein serine/threonine phosphatases is shown.

Since much of the signaling of the DDR is relayed by serine and threonine phosphorylation, it is intuitive that protein serine/threonine phosphatases would negatively regulate these phosphorylation events. Indeed, there are many proteins in the DDR that are negatively regulated in this manner, although there are also specific cases in which certain phosphatases enhance the activity of proteins in the pathway (Additional file 1, Table 1 and Fig. 2).

There are two major families of protein serine/threonine phosphatases. The phosphoprotein phosphatases include PP1, PP2A, PP2B (also known as PP3 or calcineurin), PP4, PP5, PP6, and PP7. The metal-dependent protein phosphatases, which require Mg^2+ ^or Mn^2+ ^for enzymatic activity, include PP2C (reviewed in [88,89]).

## Phosphatase action on chromatin

Multiple phosphatases have been implicated in negatively regulating γ-H2AX. In yeast, deletion of any gene of the PP4-like complex HTP-C (Pph3, Psy2, and Ybl046w) increased the amount of cellular γ-H2AX. Although Pph3 deficient cells had similar rates of DSB repair and loss of γ-H2AX foci as wild-type cells, Rad9 and Rad53 (orthologs of human 53BP1 and CHK2, respectively) remained active longer and this correlated with maintenance of the G2/M checkpoint [90].

Pharmacologic inhibition of PP2A or knockdown with siRNA increased the amount of total γ-H2AX, the number of γ-H2AX foci-positive cells, the intensity of the foci, and the amount of time the foci were maintained following treatment with the topoisomerase I inhibitor camptothecin [91]. Indeed, PP2A C co-localized with and bound to γ-H2AX after camptothecin treatment and the binding increased with increasing DNA damage [91]. Along similar lines, the inhibition of PP1 partially inhibited the elimination of γ-H2AX in human cells after IR [92]. Although PP1 has been shown to dephosphorylate γ-H2AX in nucleosomes in vitro, PP2A is at least 25 times more active toward γ-H2AX in monomeric form or when incorporated into nucleosomes [91,92].

In cells with both PP4C and PP2A C knocked-down, γ-H2AX levels were higher and sustained longer after camptothecin treatment as compared to control cells [93]. PP2A C and PP4C had comparable abilities to dephosphorylate γ-H2AX from mononuclosomes in vitro [93] and knockdown of PP4C and PP2A C also caused an increase in the total levels of γ-H2AX in untreated and IR-treated cells [94]. Further examination of the specific roles of PP2A and PP4 in the regulation of γ-H2AX revealed distinct roles for each phosphatase. PP4C-silenced cells, but not PP2A-silenced cells, showed an increase in γ-H2AX even in the absence of DNA damage [93]. PP2A C-silenced cells were slightly weakened in the ability to repair DSBs induced by X-rays while PP4C-silenced cells were not impaired in DNA repair. Knockdown of PP4C caused a slower decrease in γ-H2AX foci after IR but PP2A knockdown did not affect foci. PP4C primarily dephosphorylated γ-H2AX associated with chromatin rather than in the nucleoplasm, whereas PP2A did not. Finally, PP4C depleted cells delayed entry into mitosis, but did not have any problems with the initiation of the G2/M checkpoint [94].

PP6 can also negatively regulate γ-H2AX through binding to DNA-PK. The DNA-PK/PP6 catalytic subunit complex was disrupted by DNA-PK autophosphorylation and phosphorylation of PP6 [95]. Depletion of PP6 via siRNA prior to IR caused a sustained phosphorylation of H2AX with persistence of γ-H2AX and 53BP1 foci, increased radiation sensitivity, and a delayed exit from G2 [95].

Finally, Wip1 (Wild-type p53-induced phosphatase 1, also known as PPM1 D or PP2Cδ) was found to bind to H2AX, associate with the chromatin throughout the cell cycle, and co-localize with γ-H2AX in IR-induced foci [96]. Overexpression of Wip1 resulted in a significantly impaired induction of γ-H2AX after IR and UV and decreased γ-H2AX foci staining in an ATM-independent manner [96,97]. The spleen tissue of *Wip1*^-/- ^mice had higher basal levels of γ-H2AX than *Wip1*^+/+ ^mice in the absence of DNA damage as well as higher levels of γ-H2AX after IR [97]. Interestingly, overexpression of Wip1 inhibited the formation of MDC1 and 53BP1 foci [96,97]. Silencing of Wip1 also resulted in more efficient repair of the break since Wip1 inhibited both homology-directed recombination and non-homologous end joining [97].

Thus, several phosphatases (PP2A, PP4, PP1, PP6, and Wip1) participate directly or indirectly in the dephosphorylation of γ-H2AX. It is still not clear what the exact contribution of each phosphatase is but the emerging data suggest some level of redundancy as well as some context-dependent specificity. In addition, although it seems clear that serine/threonine phosphatases predominate in DDR signaling, tyrosine phosphorylation also plays a part. Recent data has shown that Eyes Absent tyrosine phosphatases are involved in the dephosphorylation of the H2AX Y142 residue which is involved in the formation of γ-H2AX foci [98].

PP2A is also involved in the regulation of Replication Protein A (RPA) which coats single stranded DNA [99]. The RPA-coated ssDNA acts as the substrate for RAD51 recombinase and mediates the DNA strand invasion during homology-directed recombination [99,100]. After treatment and removal of hydroxyurea, when RPA is phosphorylated, RPA32 is progressively dephosphorylated at T21 and S33. Inhibition of PP2A with okadaic acid or knockdown via siRNA results in persistence of RPA phosphorylation and foci [101]. An RPA mutant that could not be dephosphorylated resulted in reduced cell viability following UV or hydroxyl urea treatment and reduced DNA repair following replication stress [101].

## Regulating the proximal kinases ATM, ATR, and DNA-PK

The first evidence that PP2A might play a role in the ATM-dependent DDR came from the finding that the PP2A B subunit dissociates from the PP2A heterotrimer in the nucleus in an ATM-dependent manner after IR [102]. It was later found that inhibition of PP2A with okadaic acid or expression of a dominant-negative PP2A C increased ATM autophosphorylation at S1981 under normal conditions, but did not affect ATM activity. ATM binds to the A and C subunits, but this binding is disrupted after IR, partially in an ATM-dependent manner [103]. PP2A was able to dephosphorylate ATM S1981, but not as efficiently as Wip1 [104], described below.

Two different *Wip1 *mouse models have revealed a role for Wip1 in negatively regulating Atm [104,105]. E1A+Ras expressing *Wip1*-null mouse embryo fibroblasts (MEFs) have an increase in Atm phosphorylation at S1987 (corresponding to human ATM S1981) and an increase in Atm activity under normal conditions as well as after IR. Phosphorylation of p53 S18 (human S15), the Atm phosphorylation site, was increased slightly as well [104]. Indeed, Wip1 was found to dephosphorylate Atm at S1981, S367 and S1893 in vitro [104,105]. Splenocytes from *Wip1*^+/- ^and *Wip1*^-/- ^mice in an Eμ-myc background displayed an increase in Atm phosphorylation and p53 S18 phosphorylation [105]. Interestingly, *Wip1*^+/- ^and *Wip1*^-/- ^mice were more resistant to tumor formation and this was dependent on Atm and p53 [105].

In human cells, the overexpression of Wip1 decreased ATM autophosphorylation and ATM-dependent CHK2 phosphorylation after IR. The induction of Wip1 protein levels in control cells correlated with the decrease in phosphorylated ATM after IR. ATM phosphorylation also remained longer after IR in cells with lower levels of Wip1 [104]. Another study, however, used a tetracycline-inducible *Wip1 *and showed that the expression of Wip1 after IR did not have an effect on ATM S1981 phosphorylation [106]. This difference may be due to the different cell lines and conditions used.

In contrast to PP2A and Wip1 which suppress ATM activation, PP5 plays an important role in the activation of the DDR through ATM. Cells with decreased PP5 protein or activity exhibited a decrease in ATM autophosphorylation at S1981, a decrease in ATM kinase activity, and a phenotype of radio-resistant DNA synthesis after DNA damage, which is consistent with the phenotype of cells lacking ATM [107]. PP5-deficient MEFs displayed a defect in the G2/M checkpoint after IR, a decrease in Atm activity, and less phosphorylation of the Atm targets Chk2 and Nbs1 after DNA damage [108]. Interestingly, PP5 was found to bind to ATM, and this binding was increased after treatment with IR or neocarzinostatin (which causes DSB) [107].

In addition to its role in activating ATM, PP5 was also shown to be important for the activation of ATR. PP5 can bind to ATR after treatment with neocarzinostatin, UV, and hydroxyurea (which mimics replication stress). PP5 was necessary for the phosphorylation of CHK1 at S345, and the knockdown of PP5 inhibited the replication checkpoint after UV, inhibited the S-phase checkpoint after hydroxyurea, and decreased RPA phosphorylation and foci formation [109].

PP1, PP2A, and PP6 have all been shown to positively influence DNA-PK activity. When PP1 was added to DNA-PK complexes that were induced to autophosphorylate, the reduction in activity and disruption of the DNA-PK-DNA binding due to the autophosphorylation were reversed [110]. PP2A dephosphorylated DNA-PKcs as well as Ku70 and Ku80 leading to increased DNA-PK activity [111]. This opposes the autophosphorylation of DNA-PK that results in decreased activity of DNA-PK [112]. Camptothecin-induced DSB increased the association between PP2A and Ku proteins. PP2A dephosphorylation of DNA-PKcs and Ku increased the association between the DNA-PK proteins and this promoted DNA repair [113].

PP6 and DNA-PK form a complex in cells and binding increased after IR [114]. IR caused a translocation of DNA-PK and PP6 from the cytoplasm to the nucleus and it appears that localization of the phosphatase is dependent on the kinase and vice versa. Knockdown of PP6 almost completely abolished the increase in DNA-PK activity after IR, caused a defect in DSB repair, and resulted in clonogenic survival after IR similar to DNA-PK knockdown cells [114].

## Keeping effector kinases and mediators in check

CHK1 has been shown to be negatively regulated by multiple protein serine/threonine phosphatases. In *Schizosaccharomyces pombe*, the PP1 homologue Dis2 negatively regulates CHK1 [115]. Cells lacking Dis2 had a prolonged G2 arrest following treatment with the DSB inducer methylmethane sulfonate or the UV mimetic 4-nitroquinoline-N-oxide but DNA repair was not affected [115]. The overexpression of Dis2 caused a decrease in CHK1 activation following UV [115]. Indeed, CHK1 S345 could be dephosphorylated in vitro by Dis2 or human PP1 [106,115]. However, caution should be exercised when extrapolating data from yeast to mammalian systems as there are notable differences in the DDR, particularly at the level of CHK1 and CHK2 [116,117].

In human cells, the inhibition of PP2A induced CHK1 phosphorylation in the absence of DNA damage and also prevented CHK1 dephosphorylation after hydroxyurea removal [118]. The knockdown of PP2A increased CHK1 phosphorylation on S317 and S345 and in vitro, PP2A was able to directly dephosphorylate CHK1 [118]. In *X. laevis *egg extracts, the addition of PP2A C reversed CHK1 phosphorylation at S344 (human S345) after activation by DSB [119]. The inhibition of PP2A also enhanced CHK1 phosphorylation following activation by DSB [119].

Human CHK1 is also regulated by Wip1. Wip1 could dephosphorylate CHK1 primarily at S345 and slightly at S317 in vitro. In vivo, the overexpression of Wip1 resulted in the elimination of CHK1 phosphorylation at S345 and S317, a decrease of Cdc25C phosphorylation at S216, a decrease in Cdk1 phosphorylation at Y15, and a decrease in the S-phase and G2/M checkpoints, whereas the knockdown of Wip1 via siRNA caused the reverse effects [106]. Breast cancer cell lines overexpressing Wip1 (MCF-7, BT474, and MDAMB231) showed only a marginal increase in CHK1 S345 phosphorylation after UV as compared to the control cells (HEK and U2OS) [106]. Interestingly, *Wip1*^-/- ^MEFs in a 129/sv-C57BL6 background showed a greater increase in phosphorylation at S345 in CHK1 after IR than wild-type MEFs, whereas *Wip1^+/+ ^*and *Wip1*^-/- ^splenocytes expressing Eμ-myc did not show any difference in Chk1 S345 phosphorylation, possibly indicating a cell type specific or DNA damage specific regulation of CHK1 via Wip1 [105,106]. In summary, CHK1 phosphorylation at S317 and S345 and CHK1 activity following DNA damage are regulated by PP1, PP2A, and Wip1. These phosphatases seem to play an important role in recovery from the DDR checkpoints.

Much of the work examining the negative regulation of CHK2 has been done using the *Saccharomyces cerevisiae CHK2 *homologue Rad53. The PP2C phosphatases Ptc2 and Ptc3 as well as the type 2A phosphatase Pph3 have all been shown to negatively regulate Rad53 under different circumstances. To study DSB in yeast, a system that induces the HO endonuclease to create a single DSB at a specific locus that cannot be repaired by homologous recombination was utilized. A single break will cause a G2/M arrest and then adaptation to the checkpoint. Cells lacking Ptc2 and/or Ptc3 were defective in adaptation to an HO-induced G2/M arrest. Ptc2 and Ptc3 were required for recovery from the checkpoint as measured by Rad53 dephosphorylation and release from the G2/M checkpoint [120,121]. Ptc2 and Ptc3 were found to bind to the FHA1 domain of Rad53 and CK2 phosphorylation of Ptc2 at T376 was necessary for this interaction [120,121]. Mutation of this site prevented adaptation and recovery of the G2/M checkpoint [121]. Therefore, Ptc2- and Ptc3-mediated dephosphorylation of Rad53 results in recovery from the G2/M checkpoint.

Also in *S. cerevisiae*, deletion of the PP2A-like phosphatase Pph3 caused hypersensitivity to methylmethane sulfonate [122]. Pph3-Psy2 bound to Rad53 and dephosphorylated it directly. The Pph3-Psy2 complex was necessary for the dephosphorylation of Rad53 during recovery from the intra-S-phase checkpoint and it promoted the resumption of normal DNA synthesis following removal of methylmethane sulfonate [122].

One study attempted to differentiate the roles of Pph3, Ptc2, and Ptc3 in negatively regulating Rad53. Cells lacking Pph3 again showed hyperphosphorylation of Rad53, but Rad53 was still deactivated after methylmethane sulfonate treatment and removal [123]. Cells lacking Pph3, Ptc2, and Ptc3 have impaired Rad53 deactivation following MMS treatment, although Rad53 deactivation from replication stress was slightly delayed. Pph3 was not required for Rad53 dephosphorylation and deactivation following replication stress. Ptc2 and Ptc3 were not required for Rad53 deactivation and dephosphorylation following genotoxic stress in S-phase via hydroxyurea [123]. Thus, distinct phosphatases appear to be required for the dephosphorylation and deactivation of Rad53 following various DNA damages in yeast and the phosphatase required for recovery from replication stress has not yet been identified.

In humans, CHK2 was found to be regulated by PP2A and Wip1. Inhibition of PP2A using okadaic acid increased the phosphorylation of CHK2. Although PP2A was also found to negatively regulate ATM, the effect on CHK2 phosphorylation was ATM-independent [103]. Utilizing a yeast two-hybrid system, an in vitro binding assay, and co-immunoprecipitation, CHK2 was found to bind to PP2A A, C, and many B' subunits. CHK2 was able to phosphorylate B'γ1 and B'γ3 in vitro and this increased PP2A activity in vitro. The overexpression of B'γ3 resulted in a decrease in CHK2 phosphorylation after doxorubicin treatment, which causes DNA adducts [124]. Following cisplatin treatment, PP2A containing a B subunit was found to bind to CHK2. Inhibition of PP2A with okadaic acid or knockdown via siRNA caused an increase in CHK2 phosphorylation at T68. PP2A was found to dephosphorylate CHK2 in vitro as well [125].

Recent work from our laboratory has identified the B'α subunit of PP2A as a CHK2 binding partner [126]. B'α was found to bind to the SQ/TQ repeat region of CHK2, which is a target of ATM phosphorylation. The induction of DNA DSB by IR as well as treatment with doxorubicin caused a dissociation of the B'α and CHK2 proteins due to ATM-dependent phosphorylation of CHK2 serines 33 and 35. PP2A negatively regulates CHK2 phosphorylation at multiple sites including T68 as well as its kinase activity [126]. The subsequent reconstitution of the PP2A/CHK2 complex in later time points after damage may help to attenuate the signal [87,126,127].

Wip1 and CHK2 were found to bind in the nucleus and binding was dependent upon the CHK2 SQ/TQ domain, kinase activity, and nuclear localization signal and the Wip1 N-terminal domain [128,129]. Endogenous Wip1 and CHK2 could bind in MCF7 cells, which have a higher expression level of Wip1 and in A549 cells [128,129]. Using GST-Wip1 in a GST pull down assay with lysates of HCT15 cells stably expressing HA-CHK2, Wip1 was seen to bind to CHK2 only after IR, suggesting that CHK2 phosphorylation is important for the interaction [130]. In vitro, Wip1 could dephosphorylate CHK2 at multiple sites including T68 and this significantly reduced CHK2 kinase activity [129,130]. Overexpression of Wip1 inhibited CHK2 kinase activity in vitro, and in vivo, decreased T68 phosphorylation, caused a delay in CHK2 activation following IR, and caused a delay in G2/M arrest [129,130]. Knockdown of Wip1 resulted in sustained CHK2 T68 phosphorylation and kinase activity following IR, as well as an increase in apoptosis [129]. In the acute promyelocytic leukemia cell line NB4, arsenic trioxide could induce CHK2 T68 phosphorylation through the inhibition of Wip1 [131]. Finally, in stomach adenocarcinoma tumors, high Wip1 expression correlated with low CHK2 phosphorylation [132].

PP1 could dephosphorylate BRCA1 at the CHK2 target site S988, the ATM target site S1524, and the ATR site S1423 [133,134]. The overexpression of PP1 partially inhibited the hyperphosphorylation of BRCA1 after IR [134]. Since PP1 was shown to dephosphorylate BRCA1 at sites that have been shown to be important for BRCA1 function, it was intriguing to find that PP1 may actually act to enhance BRCA1 function. PP1 specifically binds to BRCA1 amino acids 898-901 (KVTF) [133,135]. Mutation of this site negatively affected homology-directed recombination and the localization of the HR factor Rad51 to the break site, which are functions of BRCA1 [133]. Interestingly, in human tissues, PP1 mRNA levels were significantly higher in normal tissue as compared to sporadic breast tumors [135]. This implicates PP1 in the regulation of BRCA1 function.

## Regulating the effectors

In addition to being regulated by MDM2, p53 is also negatively regulated through direct dephosphorylation by PP1 and PP2A. PP2A bound to p53 following IR and dephosphorylated S37 [136], whose phosphorylation via CHK1 and CHK2 is required for p53 transcriptional activity [77,137]. Inhibition of PP2A with okadaic acid or knock-down via siRNA caused an increase in p53 phosphorylation at S15 and an increase in p53 activity [136].

Inhibition of PP1, but not PP2A, with okadaic acid induced the p53 target Bax and, consequently, apoptosis in rabbit lens epithelial cells [138]. Inhibition of PP1 only slightly increased p53 levels but dramatically increased p53 phosphorylation at S15 and S37 which enhanced regulation of p53 targets Bcl2 and Bax. PP1 dephosphorylated p53 at S15 and S37 in vitro and in vivo and this decreased its transcriptional activity and attenuated apoptosis [139].

Importantly, Wip1 was induced in response to IR in a p53-dependent manner as a part of a negative feedback mechanism [140,141]. *Wip1*^-/- ^MEFs exhibited a slight increase in p53 phosphorylation at S15 and an increase in the levels of p53 target p21 [140]. In an in vitro assay, Wip1 could dephosphorylate p53 on S15 but not S46 [106]. After IR, *Wip1*^-/- ^MEFs had an increase in protein levels and S15 phosphorylation whereas knockdown of Wip1 with siRNA resulted in increased p53 protein levels and S15 phosphorylation. In this system, Wip1 did not have an effect on ATM or ATR after IR or UV, respectively, therefore the effect on p53 seems to be direct [106].

*Wip1*^-/- ^mice displayed a defect in T cell maturation due to sustained p53 activation [142]. *Wip1*^-/- ^MEFs had a more robust G1 arrest following IR, which may be due to increased activity of p53 [140]. Wip1 was required for recovery from arrest in G2 in a p53-dependent manner [143]. Overexpression of Wip1 decreased the S-phase and G2/M checkpoints whereas siRNA knockdown of Wip1 increased the intensity and length of the S-phase and G2/M checkpoints after UV and IR [106]. Interestingly, the Wip1 gene was found to be amplified in 11% of human breast tumors, most of which had wild-type p53 [144]. These data collectively suggest that the negative regulation of p53 by Wip1 due to abnormally high levels of Wip1 contributes to tumorigenesis.

Not only is p53 regulated by multiple protein serine/threonine phosphatases directly, but it is also regulated indirectly through the regulation of Mdm2. Mdm2 has been predicted to be a Wip1 substrate [145]. Indeed, Wip1, but not PP2A, dephosphorylated Mdm2 at S395, which is an ATM phosphorylation site [79,146]. Wip1 dephosphorylation of S395 increased the stability of Mdm2 and increased the Mdm2-p53 interaction. Wip1 inhibited Mdm2 auto-ubiquitination thereby stabilizing the protein [146].

The multiple modes of regulation of p53 and its activation via negative feedback loops signifies the importance of keeping p53 tightly regulated after DNA damage. Protein serine/threonine phosphatases play a role in regulating p53 through direct dephosphorylation via PP1, Wip1, and PP2A as well as enhancement of Mdm2 negative regulation of p53 through Wip1.

## Conclusions

In summary, recent literature has demonstrated that protein serine/threonine phosphatases have important functions both in the activation of the DDR and in its negative regulation. At least, negative regulation operates at two levels: keeping proteins in an inactive state and inactivating them following the repair of the damaged DNA. This regulation (Fig. 2) allows for the system to remain in an inactive state but poised to rapidly respond to damaged DNA as well as to recover from cell cycle arrest and resume cell cycle progression.

The data discussed here seems to reveal a scenario where multiple phosphatases target the same phosphorylation events. While this could be construed as excessive promiscuity of phosphatases we believe that a more nuanced reading of the data should be performed. First, the inherent limitations of different experimental approaches should be kept in mind. For example, genetic data (deletion or RNAi-mediated silencing) may result in changes in phosphorylation of the target protein but the effects might be indirect; pharmacologic inhibitors might have off target effects and suffer from low specificity; in vitro assays might reveal that a certain phosphatase can dephosphorylate a target but they may never co-localize in the cell. Second, although multiple phosphatases may target the same protein or phosphorylated residue they might have very specific functions depending on the type of damage, tissue type, and cell cycle compartment. Thus, although the general themes of these regulatory steps have been recently uncovered, working out the details and the dynamic behavior of the system is going to keep investigators busy for many years to come.

## Abbreviations

9-1-1 COMPLEX: Rad9-Rad1-Hus1; ATM: ataxia telangiectasia-mutated; ATR: ataxia telangiectasia and Rad3-related; ATRIP: ATR-interacting protein; DDR: DNA damage response; DNA-PK: DNA-dependent protein kinase; DSB: double stranded break; IR: ionizing radiation; MEFS: mouse embryo fibroblasts; MRN: Mre11-Rad50-Nbs1 complex; PCNA: proliferating cell nuclear antigen; RFC: replication factor C; RPA: replication protein A; UV: ultraviolet radiation.

## Competing interests

The authors declare that they have no competing interests.

## Authors' contributions

AF and AM contributed to the preparation of the manuscript and approval of its final version.

## Supplementary Material

Additional file 1 **Table 1**. Positive and negative regulators of specific phosphorylation sites of proteins in the DNA Damage Response Click here for file
