# Supplementary material for: Phosphatases in the cellular response to DNA damage
Source: Cell Commun Signal. 2010 Sep 22;8:27. doi: 10.1186/1478-811X-8-27 (PMC2954851; doi:10.1186/1478-811X-8-27)
Supplement: Additional file 1 — Table 1. Positive and negative regulators of specific phosphorylation sites of proteins in the DNA Damage Response [file 1478-811X-8-27-S1.DOC]

**Table 1** Positive and negative regulators of specific phosphorylation sites of proteins in the DNA Damage Response

| **Protein** | **Phospho site** | **Sequence** | **Positive regulators** | | | | | | **Negative regulators** | | | | | **Binding partner** |
| --- | --- | --- | --- | --- | --- | --- | --- | --- | --- | --- | --- | --- | --- | --- |
|  |  |  | **ATM** | **ATR** | **DNA-PK** | **CHK2** | **CHK1** | **PP5** | **PP1** | **PP2A** | **Wip1** | **PP4** | **PP6** |  |
| **ATM** | S1981 | EEG**S**QSTT |  |  |  |  |  |  |  |  |  |  |  |  |
| **NBS1** | S343 | PSL**S**QGVS |  |  |  |  |  |  |  |  |  |  |  |  |
| **RPA** | T21 | GGY**T**QSPG |  |  |  |  |  |  |  |  |  |  |  |  |
|  | S33 | PAP**S**QAEK |  |  |  |  |  |  |  |  |  |  |  |  |
| **CHK2** | T68 | TVS**T**QELY |  |  |  |  |  |  |  |  |  |  |  | CHK2 FHA |
|  | T383 | LMR**T**LCGT |  |  |  |  |  |  |  |  |  |  |  |  |
|  | T387 | LCG**T**PTYL |  |  |  |  |  |  |  |  |  |  |  |  |
| **CHK1** | S317 | YSS**S**QPEP |  |  |  |  |  |  |  |  |  |  |  |  |
|  | S345 | ISF**S**QPTC |  |  |  |  |  |  |  |  |  |  |  |  |
| **H2AX** | S139 | TQA**S**QEY |  |  |  |  |  |  |  |  |  |  |  | MDC1 BRCT |
| **BRCA1** | S988 | PIK**S**FVKT |  |  |  |  |  |  |  |  |  |  |  |  |
|  | S1387 | GLS**S**QSDI |  |  |  |  |  |  |  |  |  |  |  |  |
|  | S1423 | QHG**S**QPSN |  |  |  |  |  |  |  |  |  |  |  |  |
|  | S1457 | VLT**S**QKSS |  |  |  |  |  |  |  |  |  |  |  |  |
|  | S1524 | NYP**S**QEEL |  |  |  |  |  |  |  |  |  |  |  |  |
| **Claspin** | T916 | DLC**T**GKFT |  |  |  |  |  |  |  |  |  |  |  |  |
| **CDC25A** | S76 | RMG**S**SEST |  |  |  |  |  |  |  |  |  |  |  |  |
|  | S123 | RSH**S**DSLD |  |  |  |  |  |  |  |  |  |  |  |  |
|  | S178 | RQN**S**APA |  |  |  |  |  |  |  |  |  |  |  |  |
|  | S278 | PER**S**QEES |  |  |  |  |  |  |  |  |  |  |  |  |
|  | S292 | RRK**S**MSGA |  |  |  |  |  |  |  |  |  |  |  |  |
| **CDC25B** | S309 | RSP**S**MPCS |  |  |  |  |  |  |  |  |  |  |  | 14-3-3 |
| **CDC25C** | S216 | RSP**S**MPEN |  |  |  |  |  |  |  |  |  |  |  | 14-3-3 |
| **P53** | S15 | PPL**S**QETF |  |  |  |  |  |  |  |  |  |  |  |  |
|  | S20 | ETF**S**DLQK |  |  |  |  |  |  |  |  |  |  |  |  |
|  | S37 | PLP**S**QAMD |  |  |  |  |  |  |  |  |  |  |  |  |
| **E2F** | S31 | LDS**S**QIVI |  |  |  |  |  |  |  |  |  |  |  |  |
|  | S364 | RMG**S**LRAP |  |  |  |  |  |  |  |  |  |  |  |  |
| **MDM2** | S395 | EDY**S**QST |  |  |  |  |  |  |  |  |  |  |  |  |
|  | S407 | IYS**S**QEDV |  |  |  |  |  |  |  |  |  |  |  |  |
| **MDMX** | S342 | HSL**S**TSDI |  |  |  |  |  |  |  |  |  |  |  | 14-3-3 |
|  | S367 | RTI**S**APVV |  |  |  |  |  |  |  |  |  |  |  | 14-3-3 |
|  | S403 | SSE**S**QETI |  |  |  |  |  |  |  |  |  |  |  |  |

Sensors shown in purple, mediators shown in blue, signal transducing kinases shown in tan, effector kinases shown in pink, and effector proteins shown in green. Regulators target the phosphorylation site by phosphorylating (kinases) or dephosphorylating (phosphatases) it. Green and red boxes indicate that the action of the regulator is positive (i.e. activation) or negative (i.e. deactivation or suppression of activity), respectively.
